# Subtrochanteric femoral fractures and intramedullary nailing complications: a comparison of two implants

**DOI:** 10.1186/s10195-022-00645-8

**Published:** 2022-06-28

**Authors:** Michalis Panteli, James S. H. Vun, Robert M. West, Anthony Howard, Ippokratis Pountos, Peter V. Giannoudis

**Affiliations:** 1 Academic Department of Trauma & Orthopaedics, School of Medicine, University of Leeds, Clarendon Wing, Level D, Great George Street, Leeds, LS1 3EX West Yorkshire UK; 2 Leeds Institute of Rheumatic and Musculoskeletal Medicine, University of Leeds, Leeds, UK; 3 Leeds Orthopaedic & Trauma Sciences, Leeds General Infirmary, University of Leeds, Leeds, UK; 4 Leeds Institute of Health Sciences, University of Leeds, Leeds, UK; 5 NIHR Leeds Biomedical Research Unit, Chapel Allerton Hospital, Leeds, UK

**Keywords:** Subtrochanteric, Intramedullary nail, Complication(s), Non-union(s), Survivorship, Anti-rotation screw

## Abstract

**Introduction:**

Intramedullary (IM) nails are considered the ‘gold’ standard treatment for subtrochanteric femoral fractures. The incidence and risk factors for re-operation in subtrochanteric fractures remain unclear. Furthermore, no studies have compared the outcomes of different nailing systems used to treat subtrochanteric fractures in the same study population.

**Aims/objectives:**

Our study aimed to (i) investigate the cumulative incidence and factors associated with an increased risk of re-operation in subtrochanteric fractures treated with a long intramedullary (IM) nail, (ii) compare the outcomes of subtrochanteric fractures treated with long Affixus and Gamma nails, and (iii) establish whether the addition of a proximal anti-rotation screw in the Affixus nail confers any clinical benefit.

**Methods:**

A retrospective review of all adult patients admitted to a level 1 trauma centre with a subtrochanteric femur fracture treated with a long cephalomedullary IM nail over an 8-year period was conducted. Exclusion criteria were primary surgery performed at another institution, prophylactic nailing because of tumours, incomplete fractures, and patients who were lost to follow-up or died before fracture healing. Data variables were assessed for normality prior to determining the use of either parametric or non-parametric tests. Logistic regression analysis was performed to identify potential factors associated with re-operation. For the comparison between the two nail types, patients were matched into two groups of 119 each by age (10-year intervals), gender and mechanism of injury (low energy, high energy and pathological fractures). A *p-*value < 0.05 was considered significant. The Kaplan–Meier nail survival curve was used to demonstrate the survival of each nail. Data were analysed using the statistical package R (R version 3.6.0).

**Results:**

A total of 309 subtrochanteric fractures were treated with a distally locked long IM nail (re-operation rate: 22.33%) over an 8-year period. Logistic regression identified six factors associated with an increased risk of re-operation, including age < 75 years old, use of a long Gamma nail, pre-injury coxa-vara femoral neck shaft angles, an immediate post-operative reduction angle of > 10° varus, deep wound infection and non-union.

Following matching, we compared the two long cephalomedullary nailing systems used (Gamma versus Affixus nail). The only differences identified from the unadjusted analysis were a higher overall incidence of nail failure in Gamma nails due to any cause, re-operation, and impingement of the nail tip distally against the anterior femoral cortex. When we corrected for covariates, no significant differences remained evident between the two nails. From the Kaplan–Meier nail survival curves, however, the Affixus nail demonstrated better survivorship up to 5 years post-implantation in terms of nail failure and re-operation for all causes. Finally, the addition of a proximal anti-rotation screw in the Affixus nail did not seem to confer any benefit.

**Conclusion:**

We reported a 22.3% re-operation rate in our cohort of subtrochanteric fractures treated with a long IM nail. We have identified six risk factors associated with re-operation: age < 75 years old, pre-injury femoral neck shaft angle, choice of nail, varus reduction angle, fracture-related infection and non-union. The addition of a proximal anti-rotation screw in the Affixus nail did not confer any benefit.

## Introduction

Subtrochanteric fractures belong to a subgroup of proximal femur fractures located between the lesser trochanter and 5 cm distal to it [1]. Implant-related complications and fracture non-union (4 to 16%) are reported to be common in proximal femur fractures [2–4], with a high incidence of re-operation (3 to 6.7%) [5–8]. However, the exact incidence of these complications and re-operations of subtrochanteric fractures has not been investigated per se. Given the additional biomechanical advantage of a shorter lever arm and their less invasive technique of implantation in comparison to extramedullary implants (e.g. an angled blade plate), intramedullary (IM) nails are considered the ‘gold standard’ treatment [5, 9, 10].

Two of the commonest nailing systems currently used are the long Gamma nail (Gamma3 long nail; ^©^ Stryker, Kalamazoo, MI, USA) and the long Affixus nail (Affixus hip fracture nail; Zimmer Biomet™, Warsaw, IN, USA). Despite having similarities in their designs, the Affixus and Gamma nails do bear some important differences [11, 12]: their radius of curvature (1.8 m in the Affixus nail versus 1.5 m in the Gamma nail); the slightly bigger proximal nail diameter in the Affixus nail (15.6 mm versus 15.5 mm in the Gamma nail); the presence of a chamfered end, and the option of an additional proximal anti-rotation screw (adjacent to the lag screw) in Affixus nails [11].

Our study aimed to investigate the cumulative incidence and factors associated with re-operation in subtrochanteric fractures treated with a long IM nail, compare the outcomes of subtrochanteric fractures treated with Affixus versus Gamma nails, and to ascertain whether the addition of a proximal anti-rotation screw in the Affixus nail confers any benefit.

## Methods

Following institutional review board approval (registration number: LTH#2591), we conducted a retrospective analysis over an 8-year period (1 January 2009 to 31 December 2016). Inclusion criteria were skeletally mature patients presenting to our level 1 trauma centre with a subtrochanteric fracture managed with a long IM (cephalomedullary) nail. Exclusion criteria were patients with primary surgery performed at another institution, prophylactic nailing because of tumours, incomplete fractures, and patients lost to follow-up or who died before fracture healing.

**Fig. 1:**
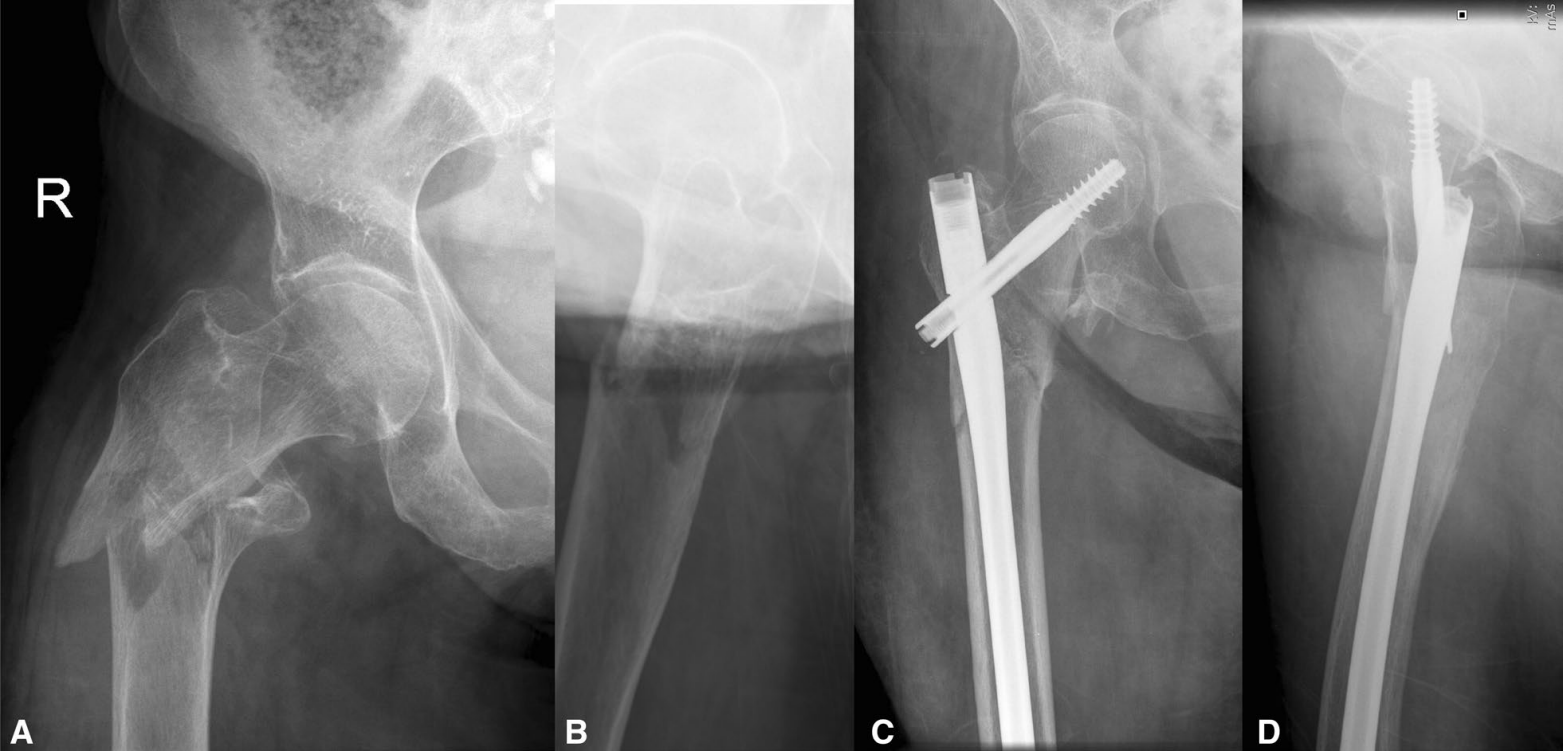
Pre-operative AP (**A**) and lateral (**B**) radiographs and post-operative AP (**C**) and lateral (**D**) radiographs of a subtrochanteric fracture managed with a Gamma nail

**Fig. 2:**
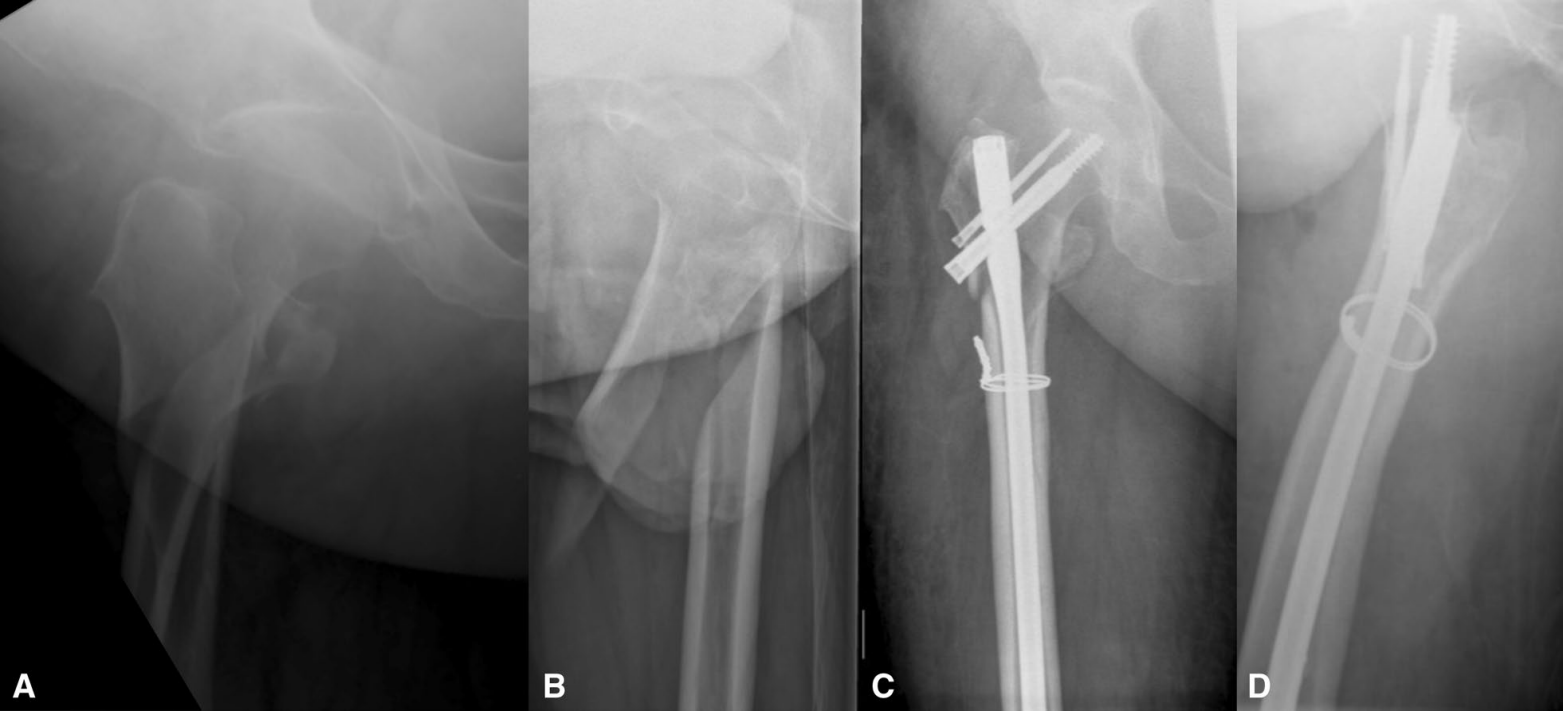
Pre-operative AP (**A**) and lateral (**B**) radiographs and post-operative AP (**C**) and lateral (**D**) radiographs of a subtrochanteric fracture managed with an Affixus nail (with a proximal anti-rotation screw and cerclage wiring to assist reduction)

Data on basic demographics, past medical history, mechanism of injury, operation characteristics, complications and outcomes were collected. The Russell–Taylor classification system was used to describe the fractures [13, 14]. Radiographic measurements were independently assessed by MP and JV; any discrepancies were resolved by the senior author (PVG). The American Society of Anaesthesiologists (ASA) Physical Status Classification was used to categorise patient comorbidities, and the Charlson Comorbidity Score (CCS) was used as a predictive tool for mortality. All patients were managed by experienced orthopaedic surgeons according to a standardised protocol. The long Gamma nail (Gamma3 long nail; ^©^ Stryker, Kalamazoo, MI, USA) (Fig. 1) was exclusively used in our institution until June 2012, with the long Affixus nail (Affixus hip fracture nail; Zimmer Biomet™, Warsaw, IN, USA) (Fig. 2) introduced and adopted thereafter. The type of nail used was changed because of a change of contracts of the hospital and not because of the performance of the nail.

Fracture healing was assessed clinically and radiologically (according to the modified radiographic union score, mRUS) [15]. Fracture-related infections were defined according to the definition provided by the AO Foundation [16–18]. We considered a failure at the lag screw junction (metalwork breakage), cut-out of the lag screw, and breakage of the distal locking screws (self-dynamisation) as nail failure. Re-operation for all causes included re-operation following nail failure, infection, removal of metalwork for any reason (i.e. impingement, post-traumatic arthritis, removal of distal screws for dynamisation of the nail) and revision for non-union. In terms of implant survivorship, we considered the first surgical re-operation of the nail for any cause as ‘non-survival’.

### Statistical analysis

Data were analysed using the statistical package R (R version 3.6.0) [19]. Data on basic demographics were presented as count (percentage) or as mean ± SD. Parametric and non-parametric data were analysed using Welch’s unpaired independent* t-*test and Pearson’s chi-square test, respectively. Following a simple logistic regression for the identification of potential associations, a revised adjusted logistic regression analysis was carried out, removing covariates in a stepwise fashion according to their likelihood-ratio chi-square *p*-value (a *p*-value < 0.05 was considered as significant). In order to reduce bias when comparing nails, patients were randomly matched by the statistical package R (using the smatch function provided by Lewer) according to age (± 5 years), gender and mechanism of injury (low energy, high energy and pathological fractures) [20]. The matching ratio was 1 and to ensure the accuracy of the results, matching was repeated using the first five seeding combinations, with no significant difference observed in the outcomes of each of the matching combinations. To further investigate the effect of the proximal anti-rotation screw used in Affixus nails, we performed a matched (as per age, gender and mechanism of injury) subgroup analysis of the patients who had an Affixus nail with or without the addition of a proximal anti-rotation screw. Finally, implant survival findings were graphically presented using Kaplan–Meier survival curves, with the log-rank test being used to identify a potential difference between the two curves.

## Results

### Re-operation in subtrochanteric fractures

A total of 309 subtrochanteric fractures treated with distally locked long IM nails fulfilled the inclusion criteria and were included in the study (Table 1). Re-operation for any cause occurred in 22.3% (*n* = 69) of all subtrochanteric fractures. In an initial unadjusted analysis, differences were noted when comparing patient demographics, medical comorbidities, and the social history of the re-operation cohort against those with no re-operation (Table 2). Larger proportion of the patients in the re-operation group were younger than 75 years old (*p* = 0.001) and active smokers (*p* = 0.002). Patients with dementia (*p* = 0.014) and a higher Charlson Comorbidity Score (*p* = 0.010) were notably more common in the cohort without re-operation. Choice of IM nailing system (long Affixus or long Gamma nail) was significantly different between the two cohorts, with the long Gamma nail used in 44.9% of all patients and accounting for 60.9% of all re-operations (*p* = 0.003). Femoral neck shaft angle (coxa valga: *p* = 0.002; coxa vara: *p* = 0.015) and degree of fracture comminution (severe: *p* = 0.013) were the only pre-operative radiographic measurements found to be significantly different between the two cohorts. In terms of post-operative radiographic measurements, the significantly different factors were a lateral cortex gap size of ≥ 5 mm (*p* = 0.002), a posterior cortex gap size of  ≥ 5 mm (*p* = 0.009) and a varus reduction angle of ≥ 5° (*p* < 0.001). The complications that were significantly different between the two cohorts were failure at the lag screw junction (*p* < 0.001), self-dynamisation (*p *< 0.001), cut-out (*p* = 0.004), non-union (*p* < 0.001), fracture-related infection (*p* < 0.001) and massive transfusion (*p* = 0.023), all of which were more common in the re-operation group.

**Table 1:** Demographics/characteristics of patients with a subtrochanteric fracture treated with a long cephalomedullary nail, stratified according to re-operation

	All patients	No re-operation	Re-operation
**Demographics**			
Total number	309	240 (77.7%)	69 (22.3%)
Age < 75 y.o.	143 (46.3%)	99 (41.2%)	44 (63.8%)
Gender			
Male	111 (35.9%)	85 (35.4%)	26 (37.7%)
Female	198 (64.1%)	155 (64.6%)	43 (62.3%)
**Injury characteristics**			
Mechanism of injury			
Low energy	244 (78.9%)	197 (82.1%)	47 (68.1%)
High energy	47 (15.2%)	32 (13.3%)	15 (21.7%)
Pathological	18 (5.8%)	11 (4.6%)	7 (10.1%)
Isolated	266 (86.1%)	206 (85.8%)	60 (87.0%)
ISS > 16	18 (5.8%)	13 (5.4%)	5 (7.2%)
Side			
Left	161 (52.1%)	119 (49.6%)	42 (60.9%)
Right	148 (47.9%)	121 (50.4%)	27 (39.1%)
Open fracture	3 (0.9%)	1 (0.4%)	2 (2.9%)
**Medical comorbidities**			
ASA			
1	28 (9.1%)	21 (8.8%)	7 (10.1%)
2	93 (30.1%)	66 (27.5%)	27 (39.1%)
3	148 (47.9%)	118 (49.2%)	30 (43.5%)
4	40 (12.9%)	35 (14.6%)	5 (7.2%)
Charlson Comorbidity Score	4.97 (2.95)	5.20 (2.92)	4.16 (2.92)
Diabetes	46 (14.9%)	39 (16.2%)	7 (10.1%)
Steroids	17 (5.5%)	15 (6.2%)	2 (2.9%)
Malignancy	72 (23.3%)	55 (22.9%)	17 (24.6%)
Dementia	44 (14.2%)	41 (17.1%)	3 (4.3%)
Osteoporosis			
Bisphosphonates pre-admission	63 (20.4%)	47 (19.6%)	16 (23.2%)
Bisphosphonates on discharge	90 (29.1%)	71 (29.7%)	19 (27.9%)
Calcium/vitamin D pre-admission	93 (30.1%)	76 (31.7%)	17 (24.6%)
Calcium/vitamin D on discharge	155 (50.2%)	126 (52.7%)	29 (42.6%)
Vitamin D loading on admission	42 (13.6%)	37 (15.5%)	5 (7.4%)
Fragility fractures before	61 (19.7%)	50 (20.8%)	11 (15.9%)
Fragility fractures after	67 (21.7%)	51 (21.2%)	16 (23.2%)
**Social history**			
Smoking	62 (20.1%)	39 (16.2%)	23 (33.3%)
Alcohol > 10 units/week	63 (20.4%)	44 (18.3%)	19 (27.5%)
Pre-operative mobility			
Independent	161 (52.1%)	120 (50.0%)	41 (59.4%)
Stick(s)/crutch(es)	98 (31.7%)	77 (32.1%)	21 (30.4%)
Frame	37 (11.9%)	32 (13.3%)	5 (7.2%)
Wheelchair/hoisted	13 (4.2%)	11 (4.6%)	2 (2.9%)
Frequent falls	85 (27.5%)	68 (28.3%)	17 (24.6%)
**Operation characteristics**			
Operation in less than 48 h	245 (79.3%)	194 (80.8%)	51 (73.9%)
Simultaneous procedures	23 (7.4%)	19 (7.9%)	4 (5.8%)
Type of nail			
Long Affixus nail	170 (55.0%)	143 (59.6%)	27 (39.1%)
Long Gamma nail	139 (44.9%)	97 (40.4%)	42 (60.9%)
Nail diameter (mm)			
9	14 (4.5%)	10 (4.2%)	4 (5.9%)
11	202 (65.4%)	156 (65.5%)	46 (67.6%)
13	90 (29.1%)	72 (30.3%)	18 (26.5%)
Open reduction	148 (47.9%)	110 (45.8%)	38 (55.1%)
Use of cerclage wires	43 (13.9%)	37 (15.4%)	6 (8.7%)
Post-op mobilisation (first 6 weeks)			
FWB	151 (48.9%)	124 (51.7%)	27 (39.1%)
PWB	76 (24.6%)	57 (23.8%)	19 (27.5%)
TTWB	50 (16.2%)	37 (15.4%)	13 (18.8%)
NWB	32 (10.4%)	22 (9.2%)	10 (14.5%)
Surgical time (min)	111.20 (43.61)	108.95 (42.50)	119 (46.75)
Anaesthetic time (min)	48.91 (22.89)	49.03 (22.93)	48.48 (22.91)
Time from induction to recovery (min)	179.75 (49.73)	177.56 (48.86)	187.36 (52.28)
Level of first surgeon			
Registrar	192 (62.1%)	148 (61.9%)	44 (63.8%)
Consultant	116 (37.5%)	91 (38.1%)	25 (36.2%)
Level of senior surgeon present			
Registrar	141 (45.6%)	137 (57.3%)	41 (59.4%)
Consultant	130 (42.1%)	102 (42.7%)	28 (40.6%)
**Complications**			
Failure at lag screw junction*	24 (7.8%)	7 (2.9%)	17 (24.6%)
Self-dynamisation	20 (6.5%)	8 (3.3%)	12 (17.4%)
Cut-out**	7 (2.3%)	1 (0.4%)	6 (8.7%)
Nail infection	5 (1.6%)	0 (0.0%)	5 (7.2%)
Peri-implant fracture	8 (2.6%)	5 (2.1%)	3 (4.3%)
Non-union***	74 (23.9%)	28 (11.7%)	46 (66.7%)
HAP/CAP	45 (14.6%)	35 (14.6%)	10 (14.5%)
UTI	43 (13.9%)	37 (15.4%)	6 (8.7%)
Fracture-related infection	11 (3.6%)	2 (0.8%)	9 (13.0%)
Revision for fracture-related infection	5 (1.6%)	0 (0%)	5 (7.4%)
CKD stage pre-operatively			
Mild	211 (68.3%)	159 (67.9%)	52 (75.4%)
Moderate/severe	92 (29.8%)	75 (32.1%)	17 (24.6%)
CKD stage post-operatively			
Mild	220 (71.2%)	166 (71.6%)	54 (79.4%)
Moderate/severe	80 (25.9%)	66 (28.4%)	14 (20.6%)
Pre-operative transfusion	22 (7.1%)	19 (7.9%)	3 (4.3%)
Post-operative transfusion (48 h)	190 (61.5%)	142 (59.2%)	48 (69.6%)
Hb drop (g/L)	43.24 (18.04)	43.17 (17.63)	43.46 (19.48)
**Radiographic measurements**			
Femoral neck shaft angle†			
Normal	209 (67.6%)	156 (65.5%)	53 (77.9%)
Coxa valga	83 (26.9%)	76 (31.9%)	7 (10.3%)
Coxa vara	14 (4.5%)	6 (2.5%)	8 (11.8%)
Number of fragments (comminution)			
Simple	90 (29.1%)	69 (28.7%)	21 (30.4%)
Moderate	147 (47.6%)	122 (50.8%)	25 (36.2%)
Severe	72 (23.3%)	49 (20.4%)	23 (33.3%)
Isolated subtrochanteric extension	50 (16.2%)	38 (15.8%)	12 (17.4%)
Distal extension****	116 (37.5%)	91 (37.9%)	25 (36.2%)
Lesser trochanter fracture	202 (65.4%)	157 (65.4%)	45 (65.2%)
Medial calcar comminution	22 (7.1%)	16 (6.7%)	6 (8.7%)
Lateral cortex gap size (mm)			
≤ 4	183 (59.2%)	156 (65.0%)	27 (39.1%)
5–9	86 (27.8%)	59 (24.6%)	27 (39.1%)
≥ 10	40 (12.9%)	25 (10.4%)	15 (21.7%)
Medial cortex gap size (mm)			
≤ 4	208 (67.3%)	169 (70.4%)	39 (56.5%)
5–9	69 (22.3%)	49 (20.4%)	20 (29.0%)
≥ 10	32 (10.4%)	22 (9.2%)	10 (14.5%)
Anterior cortex gap size (mm)			
≤ 4	199 (64.4%)	160 (66.7%)	39 (56.5%)
5–9	61 (19.7%)	49 (20.4%)	15 (21.7%)
≥ 10	46 (14.9%)	31 (12.9%)	15 (21.7%)
Posterior cortex gap size (mm)			
≤ 4	234 (75.7%)	193 (80.4%)	41 (59.4%)
5–9	57 (18.4%)	37 (15.4%)	20 (29.0%)
≥ 10	18 (5.8%)	10 (4.2%)	8 (11.6%)
Reduction angle grouped ‡ (degrees)			
Valgus 5–varus 5	218 (70.6%)	184 (76.7%)	34 (49.3%)
Valgus > 5	17 (5.5%)	13 (5.4%)	4 (5.8%)
Varus 5–10	56 (18.1%)	35 (14.6%)	21 (30.4%)
Varus > 10	18 (5.8%)	8 (3.3%)	10 (14.5%)
Anti-rotation screw	101 (32.7%)	84 (35.0%)	17 (24.6%)
TAD (mm)			
< 25	271 (87.7%)	214 (89.5%)	57 (82.6%)
≥ 25	37 (11.9%)	25 (10.5%)	12 (17.4%)
Distal locking (number of screws)			
1	11 (3.6%)	11 (4.6%)	0 (0.0%)
2	298 (96.4%)	229 (95.4%)	69 (100.0%)
Method of locking			
Static locking	201 (65.0%)	158 (66.1%)	43 (62.3%)
Secondary dynamisation	105 (33.9%)	79 (33.1%)	26 (37.7%)
Dynamic	2 (0.6%)	2 (0.8%)	0 (0.0%)
Distance of tip of the nail from centre (AP) (mm)			
−4 to 4	196 (63.4%)	154 (64.4%)	42 (60.9%)
Lateral ≥ 5	63 (20.4%)	51 (21.3%)	12 (17.4%)
Medial ≥ 5	49 (15.9%)	34 (14.2%)	15 (21.7%)
Distance of tip of the nail from centre (LAT) (mm)			
−4 to 4	244 (78.9%)	187 (78.2%)	57 (82.6%)
Anterior ≥ 5	58 (18.8%)	47 (19.7%)	11 (15.9%)
Posterior ≥ 5	6 (1.9%)	5 (2.1%)	1 (1.4%)
Distance of tip of the nail from knee (mm)			
< 10	2 (0.6%)	2 (0.8%)	0 (0.0%)
10 to 19	23 (7.4%)	15 (6.3%)	8 (11.6%)
20–29	96 (31.1%)	79 (33.1%)	17 (24.6%)
≥ 30	187 (60.5%)	143 (59.8%)	44 (63.8%)
Nail/canal ratio	0.83 (0.07)	0.82 (0.08)	0.83 (0.07)
**Hospital stay/mortality**			
HDU/ICU stay	32 (10.4%)	25 (10.4%)	7 (10.1%)
Total length of hospital stay (days)	21.76 (19.58)	22.24 (19.13)	20.09 (21.13)
Died within a year	17 (5.5%)	14 (5.8%)	3 (4.3%)

Dichotomous variables are presented as absolute numbers (percentages) of the positive event

Continuous variables are presented as mean (SD)

^*^ Seven patients in the “no re-operation” group had a failure of the nail at the lag screw junction; this happened at a later stage following the primary operation (> 6 months), and patients either had minimal or no symptoms or were unfit for an operation; therefore, conservative management was deemed appropriate

^**^ One patient presenting with lag screw cut-out was unfit for an operation and was managed conservatively

^***^ Even though 28 patients in the “no re-operation” group had an established non-union, they were managed conservatively because they had minimal or no symptoms or were unfit for an operation

^****^ More than 5 cm distal to the lesser trochanter

^†^ Femoral neck shaft angle: pre-injury angles, assumed to be the same and measured from the opposite uninjured hip. In cases of bilateral fractures, measurements were taken from the radiographs closest to the date of injury

^‡^ Reduction angle: measured in the immediate post-operative period

ISS: Injury Severity Score; ASA: American Society of Anaesthesiologists classification; DEXA: dual-energy X-ray absorptiometry; FWB: full weight bearing; PWB: partial weight bearing; TTWB: toe-touch weight bearing; NWB: non-weight bearing; HAP: hospital acquired pneumonia; CAP: community acquired pneumonia; UTI: urinary tract infection; CKD: chronic kidney disease; DVT: deep vein thrombosis; VTE: venous thromboembolism; AO: Arbeitsgemeinschaft für Osteosynthesefragen; OTA: Orthopaedic Trauma Association; TAD: tip apex distance; AP: anterior–posterior view; LAT: lateral view; HDU: high dependency unit; ICU: intensive care unit

**Table 2:** Unadjusted associations with re-operation

	Unadjusted OR (95% CI)	*p*-value
**Demographics**		
Age < 75 y.o.	2.50 (1.45–4.35)	0.001
**Medical comorbidities**		
Charlson Comorbidity Score	0.88 (0.80–0.97)	0.010
Dementia	0.22 (0.07–0.74)	0.014
**Social history**		
Smoking	2.58 (1.40–4.73)	0.002
**Operation characteristics**		
Type of nail		
Long Affixus nail	Ref.	Ref.
Long Gamma nail	2.29 (1.33–3.97)	0.003
**Radiographic measurements**		
Femoral neck shaft angle^a^		
Normal	Ref.	Ref.
Coxa valga	0.27 (0.12–0.62)	0.002
Coxa vara	3.92 (1.30–11.83)	0.015
Number of fragments (comminution)		
Simple	1.49 (0.77–2.85)	0.234
Moderate	Ref.	Ref.
Severe	2.29 (1.19–4.41)	0.013
Lateral cortex gap size (mm)		
≥ 4	Ref.	Ref.
5–9	2.64 (1.43–4.87)	0.002
≥ 10	3.47 (1.62–7.41)	0.001
Posterior cortex gap size (mm)		
≤ 4	Ref.	Ref.
5–9	2.54 (1.34–4.83)	0.004
≥ 10	3.77 (1.40–10.12)	0.009
Reduction angle grouped^b^ (degrees)		
Valgus 5–varus 5	Ref.	Ref.
Valgus > 5	1.67 (0.51–5.41)	0.397
Varus 5–10	3.25 (1.69–6.24)	< 0.001
Varus > 10	6.76 (2.49–18.37)	< 0.001
**Complications**		
Failure at lag screw junction	2.39 (0.47–5.03)	< 0.001
Self-dynamisation	6.11 (2.38–15.63)	< 0.001
Cut-out	22.76 (2.69–192.52)	0.004
Non-union	15.14 (8.01–28.63)	< 0.001
Fracture-related infection	17.85 (3.76–84.79)	< 0.001
Massive transfusion	7.32 (1.31–40.87)	0.023

^a^ Femoral neck shaft angle: measured in the opposite uninjured hip. In cases of bilateral fractures, measurements were taken from the radiographs closest to the date of injury

^b^ Reduction angle: measured in the immediate post-operative period

Following logistic regression analysis (Table 3) and adjusting for covariates, we found that (i) age < 75 years old (OR 3.00, *p* = 0.004), (ii) use of long Gamma nail (OR 2.44, *p* = 0.016), (iii) pre-injury coxa-vara (OR 4.77, *p* = 0.018) femoral neck shaft angles, (iv) immediate post-operative reduction angle of > 10° varus (OR 4.62, *p* = 0.018), (v) fracture-related infection (OR 10.65, *p* = 0.010) and (vi) non-union (OR 17.36, *p* < 0.001) were the only factors associated with re-operation.

**Table 3:** Multivariate models demonstrating associations with re-operation following a subtrochanteric fracture

	Adjusted OR (95% CI)	*p*-value
Age < 75 years old	3.00 (1.42–6.33)	0.004
Type of nail (long Gamma nail)	2.44 (1.18–5.03)	0.016
Coxa-vara femoral neck shaft angle^a^	4.77 (1.31– 17.4)	0.018
Reduction angle^b^ of > 10° varus	4.62 (1.30–16.45)	0.018
Fracture-related infection	10.65 (1.76–64.41)	0.010
Non-union	17.36 (8.19–36.81)	< 0.001

^a^Femoral neck shaft angle: pre-injury angles, assumed to be the same and measured from the opposite uninjured hip. In cases of bilateral fractures, measurements were taken from the radiographs closest to the date of injury

^b^Reduction angle: measured in the immediate post-operative period

### Does the choice of nailing system affect treatment outcome?

**Fig. 3:**
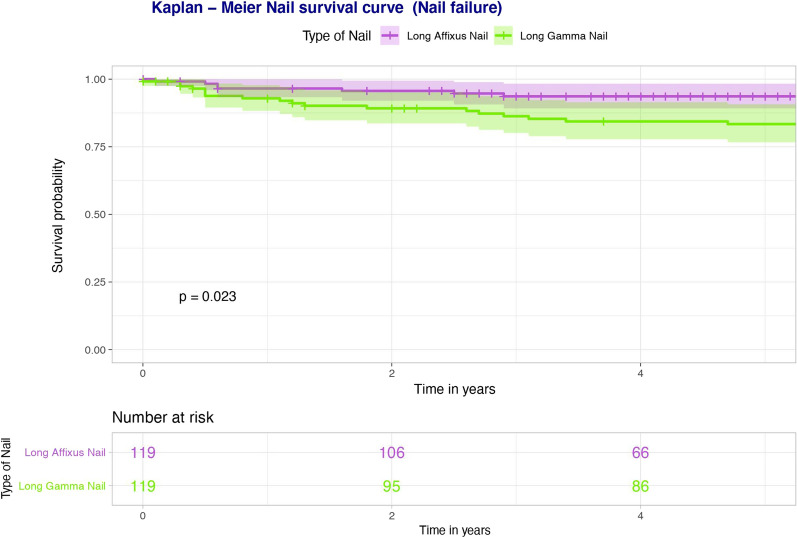
Kaplan–Meier survival curve of re-operation due to nail-failure only. The height of each KM curve represents the 95% confidence interval for each nail

**Fig. 4:**
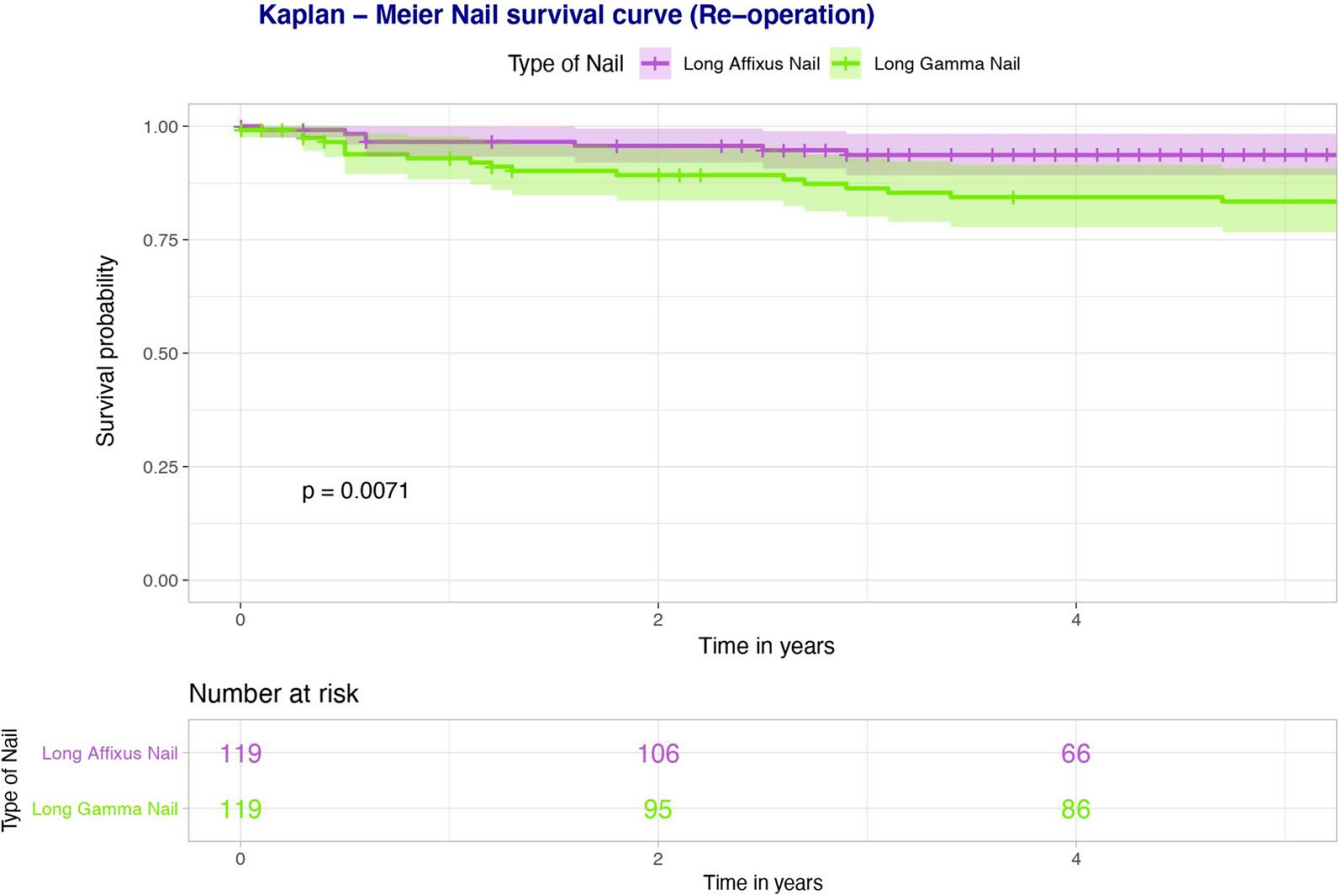
Kaplan–Meier survival curve of re-operation secondary to any cause. The height of each KM curve represents the 95% confidence interval for each nail

Basic demographics, injury characteristics, medical comorbidities, operation characteristics, radiographic measurements, complications, length of stay and mortality in a matched cohort (119 patients in each group) of patients who had long Affixus and long Gamma nails, respectively, are illustrated in Table 4. In our initial unadjusted analysis, (i) re-operation (*p* = 0.003), (ii) impinging on the anterior femoral cortex distally (*p* < 0.001) and (iii) nail failure secondary to any cause (*p* = 0.015) were the only clinical factors found to be statistically significantly different between the two nails (Table 5). Multivariate logistic regression analysis, however, yielded no statistically significant differences between the two nails. Kaplan–Meier analysis of the matched cohorts based upon (i) re-operation for nail failure only (Fig. 3) and (ii) re-operation for all causes (Fig. 4) demonstrated better survivorship in the long Affixus nail group over the long Gamma nail group (nail failure: *p* = 0.023; re-operation for all causes: *p* = 0.007).

**Table 4:** Operative characteristics and radiographic measurements (pre-operative and post-operative), stratified according to the type of nail used

	All patients	Long Gamma nail	Long Affixus nail
**Demographics**			
Total number	238	119	119
Age (years)	73.40 (16.81)	73.02 (16.86)	73.78 (16.82)
Gender			
Male	84 (35.5%)	42 (35.3%)	42 (35.3%)
Female	154 (64.7%)	77 (64.7%)	77 (64.7%)
**Injury characteristics**			
Mechanism of injury			
Low energy	200 (84.0%)	100 (84.0%)	100 (84.0%)
High energy	4 (1.7%)	2 (1.7%)	2 (1.7%)
Pathological	34 (14.3%)	17 (14.3%)	17 (14.3%)
Isolated	205 (86.1%)	103 (86.6%)	102 (85.7%)
ISS > 16	12 (5.0%)	7 (5.9%)	5 (4.2%)
Side			
Left	119 (50%)	58 (48.7%)	61 (51.3%)
Right	119 (50%)	61 (51.3%)	58 (48.7%)
Open fracture	2 (0.8%)	1 (0.8%)	1 (0.8%)
Russell–Taylor classification			
1A	68 (28.6%)	29 (24.4%)	39 (32.8%)
1B	79 (33.2%)	42 (35.3%)	37 (31.1%)
2A	11 (4.6%)	6 (5.0%)	5 (4.2%)
2B	80 (33.6%)	42 (35.3%)	38 (31.9%)
**Medical comorbidities**			
ASA			
1	19 (7.9%)	8 (6.7%)	11 (9.2%)
2	69 (28.9%)	36 (30.3%)	33 (27.7%)
3	114 (47.9%)	55 (46.2%)	59 (49.6%)
4	36 (15.1%)	20 (16.8%)	16 (13.4%)
Charlson Comorbidity Score	10.12 (4.30)	5.21 (2.80)	4.91 (2.88)
Diabetes	37 (15.5%)	15 (12.6%)	22 (18.5%)
Steroids	10 (4.2%)	4 (3.4%)	6 (5.0%)
Malignancy	50 (21.0%)	29 (24.4%)	21 (17.6%)
Dementia	39 (16.4%)	22 (18.5%)	17 (14.3%)
Osteoporosis			
Bisphosphonates pre-admission	45 (18.9%)	24 (20.2%)	21 (17.6%)
Bisphosphonates on discharge	70 (29.4%)	43 (36.4%)	27 (22.7%)
Calcium/vitamin D pre-admission	73 (30.7%)	35 (29.4%)	38 (31.9%)
Calcium/vitamin D on discharge	129 (54.2%)	72 (61.0%)	57 (47.9%)
Vitamin D loading on admission	32 (13.4%)	3 (2.5%)	29 (24.4%)
Fragility fractures before	49 (20.6%)	17 (14.3%)	32 (26.9%)
Fragility fractures after	52 (21.8%)	34 (28.6%)	18 (15.1%)
**Social history**			
Smoking	49 (20.6%)	25 (21.0%)	24 (20.2%)
Alcohol > 10 units/week	45 (18.9%)	21 (17.6%)	24 (20.2%)
Pre-operative mobility			
Independent	119 (50.0%)	62 (52.1%)	57 (47.9%)
Stick(s)/crutch(es)	80 (33.6%)	40 (33.6%)	40 (33.6%)
Frame	30 (12.6%)	13 (10.9%)	17 (14.3%)
Wheelchair/hoisted	9 (3.8%)	4 (3.4%)	5 (4.2%)
Frequent falls	73 (30.7%)	44 (37.0%)	29 (24.4%)
**Operation characteristics**			
Operation in less than 48 h	187 (78.6%)	79 (66.4%)	108 (90.8%)
Simultaneous procedures	20 (8.4%)	10 (8.4%)	10 (8.4%)
Nail diameter (mm)			
9	7 (2.9%)	0 (0.0%)	7 (5.9%)
11	153 (64.3%)	82 (70.1%)	71 (60.2%)
13	75 (31.5%)	35 (29.9%)	40 (33.9%)
Open reduction	117 (49.2%)	59 (49.6%)	58 (48.7%)
Use of cerclage wires	35 (14.7%)	17 (14.3%)	18 (15.1%)
Post-op mobilisation (first 6 weeks)			
FWB	114 (47.9%)	52 (43.7%)	62 (52.1%)
PWB	61 (25.6%)	33 (27.7%)	28 (23.5%)
TTWB	38 (15.9%)	22 (18.5%)	16 (13.4%)
NWB	25 (10.5%)	12 (10.1%)	13 (10.9%)
Surgical time (min)	109.26 (42.94)	104.06 (46.38)	114.51 (38.65)
Anaesthetic time (min)	49.30 (24.53)	45.78 (28.15)	52.85 (19.72)
Time from induction to recovery (min)	178.00 (49.82)	171.44 (54.65)	184.63 (43.66)
Level of first surgeon			
Registrar	150 (63.0%)	72 (60.5%)	78 (66.1%)
Consultant	87 (36.6%)	47 (39.5%)	40 (33.9%)
Level of senior surgeon present			
Registrar	140 (58.8%)	68 (57.1%)	72 (61.0%)
Consultant	97 (40.8%)	51 (42.9%)	46 (39.0%)
**Complications**			
Re-operation	50 (21.0%)	34 (28.6%)	16 (13.4%)
Nail failure (any cause)	25 (10.5%)	18 (15.1%)	7 (5.9%)
Failure at lag screw junction**	18 (7.6%)	11 (9.2%)	7 (5.9%)
Self-dynamisation	13 (5.5%)	9 (7.6%)	4 (3.4%)
Cut-out	6 (2.5%)	5 (4.2%)	1 (0.8%)
Nail infection	2 (0.8%)	1 (0.8%)	1 (0.8%)
Peri-implant fracture	8 (3.4%)	5 (4.2%)	3 (2.5%)
Non-union	53 (22.3%)	30 (25.2%)	23 (19.3%)
HAP/CAP	40 (16.8%)	17 (14.3%)	23 (19.3%)
UTI	35 (14.7%)	12 (10.1%)	23 (19.3%)
Wound infection			
Superficial	8 (3.4%)	4 (3.4%)	4 (3.4%)
Deep	6 (2.5%)	3 (2.5%)	3 (2.5%)
Washout/revision for Infection	4 (1.7%)	3 (9.1%)	1 (6.2%)
CKD stage pre-operatively			
Mild	163 (68.5%)	72 (62.6%)	91 (77.1%)
Moderate/severe	70 (29.4%)	43 (37.4%)	27 (22.9%)
CKD stage post-operatively			
Mild	170 (71.4%)	84 (74.3%)	86 (73.5%)
Moderate/severe	60 (25.2%)	29 (25.7%)	31 (26.5%)
Pre-operative transfusion	16 (6.7%)	11 (9.2%)	5 (4.2%)
Post-operative transfusion (48 h)	148 (62.2%)	76 (63.9%)	72 (60.5%)
Hb drop (g/L)	42.83 (17.80)	44.9 (19.09)	40.84 (16.30)
**Radiographic measurements**			
Femoral neck shaft angle^a^			
Normal	159 (66.8%)	80 (67.2%)	79 (66.9%)
Coxa valga	67 (28.2%)	32 (26.9%)	35 (29.7%)
Coxa vara	11 (4.6%)	7 (5.9%)	4 (3.4%)
Number of fragments (comminution)			
Simple	60 (25.2%)	31 (26.1%)	29 (24.4%)
Moderate	125 (52.5%)	57 (47.9%)	68 (57.1%)
Severe	53 (22.3%)	31 (26.1%)	22 (18.5%)
Only subtrochanteric extension	32 (13.4%)	14 (11.8%)	18 (15.1%)
Atypical	15 (6.3%)	8 (6.7%)	7 (5.9%)
Pathological	3 (1.3%)	2 (1.7%)	1 (0.8%)
Distal extension	94 (39.5%)	53 (44.5%)	41 (34.5%)
Greater trochanter fracture	24 (10.1%)	10 (8.4%)	14 (11.8%)
Lesser trochanter fracture	163 (68.5%)	80 (67.2%)	83 (69.7%)
Medial calcar comminution	18 (7.6%)	9 (7.6%)	9 (7.6%)
Lateral cortex gap size (mm)			
≤ 4	136 (57.1%)	60 (50.4%)	76 (63.9%)
5–9	68 (28.6%)	39 (32.8%)	29 (24.4%)
≥ 10	34 (14.3%)	20 (16.8%)	14 (11.8%)
Medial cortex gap size (mm)			
≤ 4	156 (65.5%)	83 (69.7%)	73 (61.3%)
5–9	57 (23.9%)	25 (21.0%)	32 (26.9%)
≥ 10	25 (10.5%)	11 (9.2%)	14 (11.8%)
Anterior cortex gap size (mm)			
≤ 4	156 (65.5%)	82 (68.9%)	74 (62.2%)
5–9	52 (21.8%)	21 (17.6%)	31 (26.1%)
≥ 10	30 (12.6%)	16 (13.4%)	14 (11.8%)
Posterior cortex gap size (mm)			
≤ 4	180 (75.6%)	85 (71.4%)	95 (79.8%)
(5–9)	42 (17.6%)	23 (19.3%)	19 (16.0%)
≥ 10	16 (6.7%)	11 (9.2%)	5 (4.2%)
Reduction angle grouped^b^ (degrees)			
Valgus 5–varus 5	170 (71.4%)	84 (70.6%)	86 (72.3%)
Valgus > 5	12 (5.0%)	4 (3.4%)	8 (6.7%)
Varus 5–10	44 (18.5%)	24 (20.2%)	20 (16.8%)
Varus > 10	12 (5.0%)	7 (5.9%)	5 (4.2%)
Anti-rotation screw	74 (31.1%)	0 (0.0%)	74 (62.2%)
TAD (mm)			
< 25	210 (88.2%)	107 (89.9%)	103 (87.3%)
≥ 25	27 (11.3%)	12 (10.1%)	15 (12.7%)
Distal locking (number of screws)			
1	7 (2.9%)	4 (3.4%)	3 (2.5%)
2	231 (97.1%)	115 (96.6%)	116 (97.5%)
Method of locking			
Static locking	155 (65.1%)	72 (60.5%)	83 (69.7%)
Secondary dynamisation	81 (34.0%)	46 (38.7%)	35 (29.4%)
Dynamic	2 (0.8%)	1 (0.8%)	1 (0.8%)
Distance of tip of the nail from centre (AP) (mm)			
− 4 to 4	148 (62.2%)	71 (59.7%)	77 (64.7%)
Lateral ≥ 5	50 (21.0%)	27 (22.7%)	23 (19.3%)
Medial ≥ 5	40 (16.8%)	21 (17.6%)	19 (16.0%)
Distance of tip of the nail from centre (LAT) (mm)			
− 4 to 4	187 (78.6%)	90 (75.6%)	97 (81.5%)
Anterior ≥ 5	46 (19.3%)	27 (22.7%)	19 (16.0%)
Posterior ≥ 5	5 (2.1%)	2 (1.7%)	3 (2.5%)
Distance of tip of the nail from knee (mm)			
< 10	1 (0.4%)	1 (0.8%)	0 (0.0%)
10 to 19	18 (7.6%)	6 (5.0%)	12 (10.1%)
20–29	79 (33.2%)	35 (29.4%)	44 (37.0%)
≥ 30	140 (58.8%)	77 (64.7%)	63 (52.9%)
Touching anterior cortex	74 (31.1%)	45 (37.8%)	29 (24.4%)
Nail/canal ratio	0.82 (0.08)	0.83 (0.08)	0.82 (0.07)
**Hospital stay/mortality**			
HDU/ICU stay	25 (10.5%)	10 (8.4%)	15 (12.6%)
Total length of hospital stay (days)	23.65 (21.29)	23.74 (20.19)	23.56 (22.42)
Died within a year	14 (5.9%)	10 (8.4%)	4 (3.4%)

Dichotomous variables are presented as absolute numbers (percentages) of the positive event

Continuous variables are presented as mean (SD)

^a^Femoral neck shaft angle: pre-injury angles, assumed to be same and measured from the opposite uninjured hip. In cases of bilateral fractures, measurements were taken from the radiographs closest to the date of injury

^b^Reduction angle: measured in the immediate post-operative period

**Table 5:** Unadjusted associations of long Affixus vs long Gamma nails

	Unadjusted OR (95% CI)	*p*-value
**Complications**		
Re-operation	0.44 (0.25–0.75)	0.003
Nail failure (any cause)	0.37 (0.17–0.82)	0.015
**Radiographic measurements**		
Touching anterior cortex	0.42 (0.25–0.70)	< 0.001

Long Gamma nail: control

Investigating the effect of a proximal anti-rotation screw in matched patients receiving an Affixus nail, we found the two groups (with versus without a proximal anti-rotation screw) to be comparable with regards to all variables examined. More specifically, there was no significant difference in fracture reduction, length of operation, complications including non-union, infection and nail failure, as well as revision for any cause.

## Discussion

Despite subtrochanteric fractures being commonly treated with an IM nail, evidence on the incidence and risk factors of re-operation in this subgroup of fractures is still lacking. In addition, as yet, no studies have compared the outcomes of different nailing systems used to treat subtrochanteric fractures in the same study population.

This study reported a re-operation rate of 22.3% in subtrochanteric fractures treated with a distally locked IM nail. Despite the differences in demographics, injury patterns, comorbidities and complications between patients who required a re-operation against those who did not, following an adjusted analysis, only six factors were found to be associated with re-operation. These include (i) age < 75 years old, (ii) nail type (long Gamma nail), (iii) pre-injury femoral neck shaft angle (coxa vara), (iv) a varus reduction angle > 10°, (v) fracture-related infection, and (vi) non-union.

A better way of understanding these six risk factors for re-operation would be to group them into (i) biological factors (age, infection, non-union) and (ii) anatomical factors (pre-injury femoral neck shaft angle, implant choice, reduction angle). Younger patients are more likely to sustain high-energy injuries, which often result in comminuted fracture patterns and other insults to the zone of injury (e.g. open fracture, vascular injury) [21]. All these deleterious factors ultimately subject the fracture to a higher risk of non-union [21]. Unless the fracture non-union or its causative factor (such as a fracture-related infection) is addressed and treated, the repetitive cyclic loading would, over time, exceed the failure load of the IM nail, resulting in nail failure [22, 23].

From a mechanical perspective, the pre-injury femoral neck shaft angle, reduction angle and nail type were all risk factors for nail failure leading to re-operation. A varus femoral neck shaft angle, whether congenital or secondary to malreduction, subjects the nail/lag screw junction to significant loading and bending stress, risking nail failure [22].

Comparing the use of long Affixus and Gamma nails in subtrochanteric fractures, outcomes of subtrochanteric fractures treated with the two nails in question have been reported in only a few studies [24, 25]. Most studies have only reported on the outcomes of Gamma nail use in intertrochanteric fractures [25–28]. Surgical time (Gamma nail: 104.1 min; Affixus nail: 114.5 min) and length of stay (Gamma nail: 23.7 days; Affixus nail: 23.6 days) were comparable between the two nails in our study group. This finding is similar to that of Persiani et al., who, to our knowledge, performed the only study that compared the use of Affixus and Gamma nails in the treatment of trochanteric fractures [26]. Mortality rates at 1 year were not significantly different between the two nail groups. On the other hand, the rates of re-operation, nail failure and touching of the anterior cortex in our study cohort were lower in the Affixus nail group when compared against the Gamma nail group (re-operation: *p* = 0.003; nail failure: *p* = 0.015; touching of anterior cortex: *p *< 0.001) (Table 5).

Complications were common with both nails. The two commonest complications in our patient cohort were non-union and fracture-related infection (Table 4). The incidence rates of non-union (25.2% in the Gamma nail group, 19.3% in the Affixus nail group) and fracture-related infection (2.5% in both the Gamma and Affixus nail groups) in our cohort of patients with subtrochanteric fractures were much higher than those reported in proximal femur fractures (non-union: 6.3%; fracture-related infection: 1.1%) [25, 27, 28]. The smaller (albeit not statistically significantly smaller) non-union rates in the Affixus group could be explained by the better lateral cortical reduction demonstrated with this nailing system (Table 4). Failure at the lag screw junction was another common complication, occurring at rates of 9.2% and 5.9% in the Gamma and Affixus nail groups, respectively. The cut-out rate in our Affixus nail group (0.8%) was slightly less than for proximal femur fractures treated with cephalomedullary nails (1.1 to 2.7%) [25, 28], whereas it was notably higher amongst the patients in the Gamma nail group (4.2%). Lastly, peri-implant fractures occurred in 4.2% and 2.5% of the Gamma and Affixus groups, respectively. Individual nail complications were not statistically different between the two nails, as previously mentioned, but the collective risk of nail failure due to all nail complications was significantly lower in the Affixus nail group (5.9% in the Affixus vs 15.1% in the Gamma nail group; *p* = 0.02).

The Affixus nail was noted to have superior performance, based on our 5-year Kaplan–Meier survival curve analyses (Figs. 3 and 4). The reasons for the improved survivorship of Affixus nails ought, however, to be interpreted with care. It is noteworthy that the Affixus nail was used during the second half of the study, when improvements had been made to the care pathway of patients with fragility fractures (e.g. orthogeriatric input, time to surgery of less than 48 h from the time of injury). Therefore, the improved survivorship of Affixus nails could well be multifactorial, and not attributable to just the nailing system alone.

Lastly, our study has addressed several controversial topics surrounding the use of a proximal anti-rotation screw in the Affixus nail. Implantation of the additional proximal anti-rotation screw did not lead to any statistically significantly different rates of complications, including non-union, infection and nail failure, as well as revision for any cause, therefore allaying the concerns over the additional surgical step and potential complications caused by the implantation of this additional proximal anti-rotation screw, such as the ‘Z-effect’, whereby the inferior lag screw migrates laterally and the superior screw migrates medially, leading to perforation of the femoral head by the superior screw [29].

To date, this study represents the largest series reporting on the incidence and associations of re-operation in subtrochanteric fractures treated with a long cephalomedullary nail. Furthermore, this study also compared the use of Gamma and Affixus nails in subtrochanteric femur fractures, and the effects of a proximal anti-rotation screw in the Affixus nail. One of the strengths of our study design lies in our inclusion criteria, which were generally more inclusive, with no restrictions imposed upon age or comorbidity, therefore allowing for a more accurate representation of the outcomes of these nails when used to treat subtrochanteric fractures. A further strength lies in the random matching of patients by age, gender and mechanism of injury (low energy, high energy and pathological fractures), thus removing any inherent risk of selection bias during statistical analysis. The retrospective nature of our study is a limitation, as data collection could be subject to bias. The analysis of fracture and radiological features could also be subject to inter- and intra-observer bias, which we hope to have addressed by utilising two assessors to analyse the results. Formal intra- and inter-observer reliability testing would help to reduce this risk. Additionally, measurements such as the neck shaft angle can be difficult to capture on plain radiographs, as flexion, abduction or external rotation of the hip can affect them. We assume, however, that the error was similar in the two groups, and therefore the effect of this error is minimised. Another limitation lies in the fact that the Affixus nail and the Gamma nail were used over different periods of the study. A prospective, randomised controlled trial would have been a superior model, providing a more accurate comparison of the survivorship between the two nailing systems.

## Conclusion

Our study reported a 22.3% re-operation rate amongst subtrochanteric fractures treated with a distally locked long IM nail. We have demonstrated that age < 75 years old, a pre-injury coxa-vara femoral neck shaft angle, choice of nail, a varus reduction angle, a fracture-related infection and non-union are factors associated with an increased risk of re-operation. The addition of a proximal anti-rotation screw in the Affixus nail did not confer any benefit. Given the high non-union rate of this subtype of fracture, to provide patients with the best chances of a successful outcome, we advise careful consideration of these factors when treating subtrochanteric femur fracture patients with an IM nail. Further research is required to better understand the survivorship and demonstrate the clear advantage of one nail over the other.

## Data Availability

On request to the corresponding author.
